# Response of Polygenic Traits Under Stabilizing Selection and Mutation When Loci Have Unequal Effects

**DOI:** 10.1534/g3.115.017970

**Published:** 2015-03-31

**Authors:** Kavita Jain, Wolfgang Stephan

**Affiliations:** * Theoretical Sciences Unit, Jawaharlal Nehru Centre for Advanced Scientific Research, Jakkur P.O., Bangalore, 560064 India; † Section of Evolutionary Biology, Department of Biology, Ludwig-Maximilians University of Munich, Planegg-Martinsried, Germany

**Keywords:** polygenic selection, mutation, unequal effects, dynamics, genetic variance, rapid adaptation

## Abstract

We consider an infinitely large population under stabilizing selection and mutation in which the allelic effects determining a polygenic trait vary between loci. We obtain analytical expressions for the stationary genetic variance as a function of the distribution of effects, mutation rate, and selection coefficient. We also study the dynamics of the allele frequencies, focusing on short-term evolution of the phenotypic mean as it approaches the optimum after an environmental change. We find that when most effects are small, the genetic variance does not change appreciably during adaptation, and the time until the phenotypic mean reaches the optimum is short if the number of loci is large. However, when most effects are large, the change of the variance during the adaptive process cannot be neglected. In this case, the short-term dynamics may be described by those of a few loci of large effect. Our results may be used to understand polygenic selection driving rapid adaptation.

In the study of fast adaptation, polygenic selection may be more important than selection on single genes. At single genes, strong selection driving fast adaptation generally leads to the rapid fixation of beneficial alleles or at least large allele frequency shifts between populations. Classical examples of this type of adaptation are the case of industrial melanism in moths (van’t Hof *et al.* 2011) and insecticide resistance in *Drosophila* (Daborn *et al.* 2002). However, in many (if not most) cases of fast adaptation, such as in island lizards that are able to adapt very quickly to a changing vegetation [*e.g.*, revealed in an experimental evolution study by Kolbe *et al.* (2012)], small shifts of allele frequencies at many loci may be sufficient to move a phenotype toward a new optimum under changed environmental conditions.

There is a large and growing body of literature on the detection of adaptive signatures in molecular population genetics. Following pioneering work of Maynard Smith and Haigh (1974), the impact of positive selection on neutral DNA variability (selective sweeps) has attracted much interest. This theory has been applied to huge datasets that emerge from modern high-throughput sequencing. A large number of statistical tests have been developed to detect sweep signals and estimate the frequency and strength of selection (Kim and Stephan 2002; Nielsen *et al.* 2005; Pavlidis *et al.* 2010). However, most theory so far excludes the phenotypic side of the adaptive process (except for fitness). Usually, selection is simply modeled as a constant force that acts on a new allele at a single locus. This is in striking contrast to the classical phenotype-based models of adaptation that are successfully used in quantitative genetics (Barton and Keightley 2002). These models typically assume that adaptations are based on allele frequency shifts of small or moderate size at a large number of loci. Also, adaptation does not require new mutations, at least in the short term. Instead, selection uses alleles that are found in the standing genetic variation. Genome-wide data of the past few years show that this quantitative genetic view is relevant. In particular, association studies confirm that quantitative traits are typically highly polygenic. High heritabilities most probably result from standing genetic variation at a large number of loci with small individual effect. Also, local adaptation to environmental clines involves moderate frequency shifts at multiple loci (Hancock *et al.* 2010). As a consequence, there is growing evidence that the molecular scenario of sweeps only covers part of the adaptive process and needs to be revised to include polygenic selection.

Because genome-wide association studies (GWAS) yield information about the distribution of single-nucleotide polymorphisms relevant to quantitative traits (Visscher *et al.* 2012), it is important to understand the models of polygenic selection in terms of the frequency changes of molecular variants, *i.e.*, in terms of population genetics. So far, however, the dynamics of only very simple polygenic models have been studied and applied to data [*e.g.*, Turchin *et al.* (2012)]. In this article, we analyze the dynamics of a quantitative trait given by a much more general model that was originally proposed by Wright (1935) and recently re-visited by de Vladar and Barton (2014). These authors consider an infinitely large population evolving under stabilizing selection and mutation. Following the observations from many empirical studies [particularly from biomedicine, see Visscher *et al.* (2012)], they assume that the effects are locus-dependent. Furthermore, they consider additivity of the effects and linkage equilibrium between loci.

Here we study this model and obtain some analytical results on the stationary genetic variance and also the dynamics of the phenotypic mean. We show that the stationary genetic variance may exhibit nonmonotonic dependence on the shape of the distribution of effects. We also study how the trajectories of the allele frequencies and the mean trait respond to a sudden environmental shift. When most effects are small, as is the case in experiments on *Drosophila* (Mackay 2004), in livestock (Hayes and Goddard 2001; Goddard and Hayes 2009), and for human height (Visscher 2008), a simple analysis shows that the magnitude of the deviation of the phenotypic mean from the optimum decays roughly exponentially with time and approaches zero over a time scale that is inversely proportional to the initial genetic variance. When most effects are large, the short-term dynamics of the mean and variance can be understood by considering a few loci with large effects.

## Model with Locus-Dependent Effects

We consider the *ℓ*-locus model recently analyzed by de Vladar and Barton (2014) where each locus is biallelic. The + allele at site *i* has frequency $p_{i}$ whereas the − allele occurs with frequency *q_i_* = 1 − *p_i_*. The effects are assumed to be additive so that the trait value is $z=\sum_{i=1}^{\ell }\text{sgn}\left(\text{i}\right)\;\gamma_{i}$, where sgn(i)=±1 denotes the sign of the genotypic value of locus *i* and $\gamma_{i}>0$ is the effect of the allele at the *i*th locus. The loci are assumed to be in linkage equilibrium so that the joint distribution of effects at the loci factorises. As a result, the *n*th cumulant $c_{n}$ of the phenotypic effect, obtained on averaging over the population distribution, can be written as the sum over the corresponding quantities at individual loci. The first three cumulants *viz*. mean *c*_1_, variance *c*_2_, and skewness *c*_3_, are given by the following (Bürger 1991):

$$c_{1}=\sum_{i=1}^{\ell }\gamma_{i}\left(p_{i}-q_{i}\right)$$ (1a)

$$c_{2}=2\sum_{i=1}^{\ell }\gamma_{i}^{2}p_{i}q_{i}$$ (1b)

$$c_{3}=2\sum_{i=1}^{\ell }\gamma_{i}^{3}\left(q_{i}-p_{i}\right)p_{i}q_{i}.$$ (1c)

The allele frequency evolves in time under selective pressure and is given by the following (Barton 1986):

$$\frac{\partial p_{i}}{\partial t}\approx p_{i}\left(t+1\right)-p_{i}\left(t\right)=\frac{p_{i}q_{i}}{2\bar{w}}\frac{\partial \bar{w}}{\partial p_{i}}\;,$$ (2)

where $\bar{w}$ is the average fitness of the population. For large *ℓ*, as the trait value *z* of an individual can be treated as a continuous variable, from (1a) and (1b), we obtain

$$\bar{w}=\int_{-\infty }^{\infty }dz\;p\left(z\right)w\left(z\right)=1-\frac{s}{2}\left(c_{2}+{\left(\Delta c_{1}\right)}^{2}\right)\approx e^{-\frac{s}{2}\left(c_{2}+{\left(\Delta c_{1}\right)}^{2}\right)}\;,$$ (3)

where the approximate equality sign holds because *s* is assumed to be small. In the above expression, $w\left(z\right)=1-\left(s/2\right){\left(z-z_{o}\right)}^{2}$ is the fitness distribution of the phenotypic trait under stabilizing selection, *z*_0_ the phenotypic optimum and $\Delta c_{1}=c_{1}-z_{o}$ the mean deviation from *z*_0_. Thus the maximum fitness (namely, one) is obtained when the population is at the phenotypic optimum and has no genetic variance. Inserting equations (1) and (3) in (2) and accounting for mutations, we obtain the following basic equation for the evolution of allele frequencies,

$$\frac{\partial p_{i}}{\partial t}=-\frac{s\gamma_{i}^{2}}{2}p_{i}q_{i}\left(2\frac{\Delta c_{1}}{\gamma_{i}}+q_{i}-p_{i}\right)+\mu \left(q_{i}-p_{i}\right),i=1,\ldots ,\ell \;,$$ (4)

where *μ* is the probability of (symmetric) mutation between the + and − allele at locus *i*. Note that the equation (1) of de Vladar and Barton (2014) is obtained by replacing *s* by 2s in the above equation.

On the right-hand side (RHS) of (4), the first term (in the first parenthesis) expressing the mean deviation from the optimum corresponds to directional selection toward the phenotypic optimum: if the mean is above (below) the optimum, the allele frequencies decrease (increase). However, once the phenotypic mean is sufficiently close to the optimum, stabilizing selection (described by the second term) takes over.

One of the difficulties in solving (4) is that it involves the mean *c*_1_, which depends on all the allele frequencies. Moreover, it has been shown that the differential equations for the cumulants do not close: each one not only involves two higher cumulants but also contains terms that cannot be written in terms of other cumulants (Barton and Turelli 1987; Bürger 1991).

## Genetic Variance in the Stationary State

In the stationary state in which the left-hand side of (4) vanishes, if the mean $c_{1}^{\text{*}}=z_{o}$, the allele frequency $p_{i}^{\text{*}}$ has three solutions, namely 1/2 and $\left(1\pm \sqrt{1-{\left(\hat{\gamma }/\gamma_{i}\right)}^{2}}\right)/2$, where $\hat{\gamma }=2\sqrt{2\mu /s}$. The latter two solutions are stable for $\gamma_{i}>\hat{\gamma }$, and therefore the allele frequency is close to fixation when the effects are large. For $\gamma_{i}<\hat{\gamma }$, the effects are small and the stationary state solution $p_{i}^{\text{*}}=1/2$ is the only stable solution for the allele frequency (de Vladar and Barton 2014). From these results, the stationary genetic variance (1b) is easily seen to be (de Vladar and Barton 2014)

$$c_{2}^{\text{*}}=\frac{4\mu }{s}n_{l}+\frac{1}{2}\sum_{\gamma_{i}<\hat{\gamma }}\gamma_{i}^{2},$$ (5)

where, for large *ℓ*, the number of effects larger than $\hat{\gamma }$ is given by $n_{l}=\ell \int_{\hat{\gamma }}^{\infty }d\gamma \;p\left(\gamma \right)$ with p(γ) being the distribution of effects. Thus the genetic variance in the stationary state can be neatly written as

$$c_{2}^{\text{*}}=\frac{\ell }{2}\left[{\hat{\gamma }}^{2}\int_{\hat{\gamma }}^{\infty }d\gamma \;p\left(\gamma \right)+\int_{0}^{\hat{\gamma }}d\gamma \;\gamma^{2}\;p\left(\gamma \right)\right],$$ (6)

where the first (second) term is the contribution from loci with large (small) effects.

The gamma distribution $p\left(\gamma \right)∼\gamma^{k-1}e^{-k\gamma /\bar{\gamma }}$ with shape parameter k>0 and mean $\bar{\gamma }$ has been used to fit the distribution of quantitative trait loci effects (Hayes and Goddard 2001). This distribution is *L*-shaped for k<1 and bell-shaped for k>1, whereas for k=1, it is an exponential function. For the gamma distribution, the stationary genetic variance (6) is given by

$$c_{2}^{\text{*}}=\frac{\ell {\hat{\gamma }}^{2}}{2}\;\left[\frac{\text{Γ}\left(k,k\gamma_{r}\right)}{\text{Γ}\left(k\right)}+\frac{\text{Γ}\left(2+k\right)-\text{Γ}\left(2+k,k\gamma_{r}\right)}{\gamma_{r}^{2}k^{2}\text{Γ}\left(k\right)}\right]\;,$$ (7)

where $\gamma_{r}=\hat{\gamma }/\bar{\gamma }$ and $\text{Γ}\left(a,b\right)=\int_{b}^{\infty }dt\;t^{a-1}e^{-t}$ is the incomplete gamma function. For the special case of exponentially distributed effects (k=1), (7) simplifies to give $c_{2}^{\text{*}}=\ell {\bar{\gamma }}^{2}\left(1-e^{-\gamma_{r}}\left(1+\gamma_{r}\right)\right)$.

For $\bar{\gamma }\gg \hat{\gamma }$, the House of Cards (HoC) variance, namely $c_{2}^{\text{*}}=\ell {\hat{\gamma }}^{2}/2=4\mu \ell /s$ (Turelli 1984), is obtained when $\hat{\gamma }$ is finite but $\bar{\gamma }\to \infty$ for all *k*. In the opposite case $\left(\bar{\gamma }\ll \hat{\gamma }\right)$, we have $c_{2}^{\text{*}}=\ell {\bar{\gamma }}^{2}\left(k+1\right)/\left(2k\right)$, which depends on the shape of the distribution of effects. For fixed mean $\bar{\gamma }$, the genetic variance increases monotonically with the scale $\hat{\gamma }$ because a larger mutation probability increases the variance. If instead the distribution mean $\bar{\gamma }$ is increased keeping $\hat{\gamma }$ fixed, the variance increases with $\bar{\gamma }$ toward the HoC value. This is because for fixed *k*, the width of the distribution increases with $\bar{\gamma }$ and therefore larger effects can be accessed.

**Figure 1:**
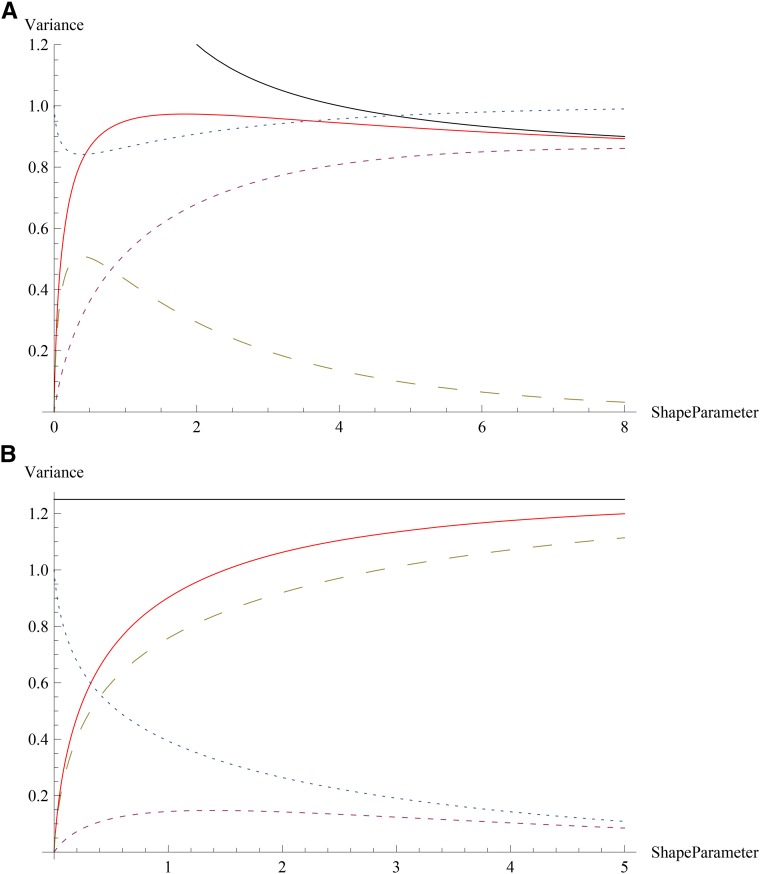
Genetic variance in the stationary state as a function of the shape parameter *k* when the effects are distributed according to the gamma function. The plot shows the total genetic variance (solid), variance caused by small effects (small dashes), large effects (large dashes), and the fraction of small effects (dotted) for (a) $\bar{\gamma }=0.04,\hat{\gamma }=0.08$ and (b) $\bar{\gamma }=0.1,\hat{\gamma }=0.05$ for ℓ=1000. The asymptotic values $\ell {\bar{\gamma }}^{2}\left(k+1\right)/\left(2k\right)$ when $\hat{\gamma }>\bar{\gamma }$ and $\ell {\hat{\gamma }}^{2}/2$ when $\hat{\gamma }<\bar{\gamma }$ are also shown (top solid curves).

The stationary genetic variance has been computed numerically for various shape parameters when $\hat{\gamma }=0.063,\bar{\gamma }=0.1$ and ℓ=1000 in de Vladar and Barton (2014). From (7), the variance for *k* = 1, 2, 10, and 100 is found to be 1.32, 1.57, 1.94, and 2, respectively, which agrees well with the numerical data in their Figure 5. To understand how the variance depends on the shape parameter, we first note that with increasing *k* (and fixed $\bar{\gamma }$), the width of the gamma distribution decreases. For large *k*, if $\bar{\gamma }>\hat{\gamma }$, the variance saturates to the HoC variance since almost all loci have large effects with narrow distributions whereas in the opposite case, most effects are small and the variance tends to $\ell {\bar{\gamma }}^{2}/2$ (see Figure 1). For small *k*, irrespective of whether $\bar{\gamma }$ is above or below $\hat{\gamma }$, we find that most effects are small. To see this, consider the fraction $f_{s}=1-\left(n_{l}/\ell \right)$ of loci with small effects which is given by

$$f_{s}=\frac{{\left(k\gamma_{r}\right)}^{k}}{\left(k-1\right)!}\int_{0}^{1}dx\;x^{k-1}e^{-k\gamma_{r}x}=1-\frac{\text{Γ}\left(k,k\gamma_{r}\right)}{\text{Γ}\left(k\right)}.$$ (8)

If $k<{\left(\gamma_{r}\right)}^{-1}$, the aforementioned equation yields $f_{s}\approx {\left(k\gamma_{r}\right)}^{k}/k!$. Then for finite *γ_r_*, when k→0, we find that $n_{s}\to \ell$ for any *γ_r_*, as claimed previously. To summarize, as shown in Figure 1, for $\hat{\gamma }<\bar{\gamma }$, the variance increases with *k* toward the HoC variance, whereas for $\hat{\gamma }>\bar{\gamma }$, both $c_{2}^{\text{*}}$ and $n_{s}$ are nonmonotonic functions of *k*.

When the effects are chosen from an exponential distribution, the fraction $f_{s}=1-e^{-\gamma_{r}}$. On eliminating $\gamma_{r}$ in favor of $f_{s}$ in (7) for k=1, we find the relative contribution of loci with small effects to the total variance to be

$$\frac{c_{2,small}^{\text{*}}}{c_{2}^{\text{*}}}=\frac{2f_{s}+\left(1-f_{s}\right)\ln\left(1-f_{s}\right)\left(2-\ln\left(1-f_{s}\right)\right)}{2f_{s}+2\left(1-f_{s}\right)\text{ln}\left(1-f_{s}\right)}\;,$$ (9)

which increases as $f_{s}/3$ for small $f_{s}$ and approaches unity as *f_s_* increases toward one. The aforementioned expression shows that if 10% of the effects are small, their contribution to the variance is merely 3%, which increases to 21% when *f_s_* is one half. To obtain an equal contribution from small and large effects, a disproportionately large fraction (∼83%) of small effects is required. We are unable to obtain an analytical expression analogous to (9) for arbitrary *k* since *f_s_* is not a simple function of *γ_r_* (see (8), shown previously). However, a numerical analysis using (7) and (8) shows that for the same value of *f_s_*, small effects contribute more to the total genetic variance as the distribution of effects gets narrower. For *f_s_* = 0.1, the relative contribution is found to be 2%, 3%, and 5% for *k* = 1/2, 1, and 2, respectively. To obtain an equal contribution from loci with small and large effects, *f_s_* = 0.89, 0.83, and 0.77 is needed for the shape parameters *k* = 1/2, 1, and 2, respectively.

## Dynamics of the Allele Frequency

We now turn to a description of the allele frequency dynamics and will consider the situation when the phenotypic optimum is suddenly shifted. As mentioned previously, due to the term $\Delta c_{1}$ on the RHS of (4), all the frequencies are coupled, which makes it hard to obtain an exact analytical solution of the allele frequency dynamics. However, under certain conditions, it is a good approximation to consider only the *c*_1_ term in (4) for the initial dynamics and the rest of the terms for long-term evolution.

To see this, we first note that because 0 < *p_i_* < 1, the mean $\left|c_{1}\left(t\right)\right|<\sum_{i}\gamma_{i}\approx \bar{\gamma }\ell$. For independent and uniformly distributed initial frequencies, as the average initial frequency is one half, the leading order contribution (in *ℓ*) to the initial mean is zero. The initial variance is, however, nonzero which gives the typical initial mean $\left|c_{1}\left(0\right)\right|∼\bar{\gamma }\sqrt{\ell }$. When the phenotypic optimum $z_{o}≲\bar{\gamma }\sqrt{\ell }$ and the number of loci is large, the initial value $\left|\Delta c_{1}\left(0\right)\right|/\gamma_{i}∼\sqrt{\ell }\gg 1$. Thus at short times, we can neglect $\left|2p_{i}-1\right|$ (which is bounded above by one) and the mutation term in comparison to the term $2\Delta c_{1}/\gamma_{i}$ in (4). At large enough crossover time *t*_×_, as explained in the following section, the mean deviation is close to zero and the reverse condition holds, *i.e.*, $2\left|\Delta c_{1}\left(t\right)\right|/\gamma_{i}\ll \left|2p_{i}-1\right|$ in (4), and we may set $\Delta c_{1}\approx 0$ for later evolution. Biologically, these considerations mean that initially the effects are weaker than the mean trait deviation, but as the population adapts due to directional selection, the deviation of the mean from the phenotypic optimum becomes smaller than the effects.

The aforementioned argument applies not only to uniformly distributed initial frequencies but in more general settings as well where $\left|c_{1}\left(0\right)\right|∼\bar{\gamma }\ell$ by replacing $\sqrt{\ell }$ by *ℓ*. Here we will focus on the dynamics of the allele frequency when the optimum is suddenly shifted to a new value $z_{f}\left(<\ell \bar{\gamma }\right)$, starting from the population which is equilibrated to a phenotypic optimum value $z_{o}$. In this situation, as the initial frequency is close to one half when $\gamma_{i}<\hat{\gamma }$, the frequency $\left|2p_{i}\left(0\right)-1\right|$ is obviously negligible compared with $\Delta c_{1}\left(0\right)/\gamma_{i}$, whereas for $\gamma_{i}>\hat{\gamma }$, $\left|2p_{i}\left(0\right)-1\right|$ is close to one because the initial frequency is close to either zero or one (de Vladar and Barton 2014).

### When most effects are small

**Figure 2:**
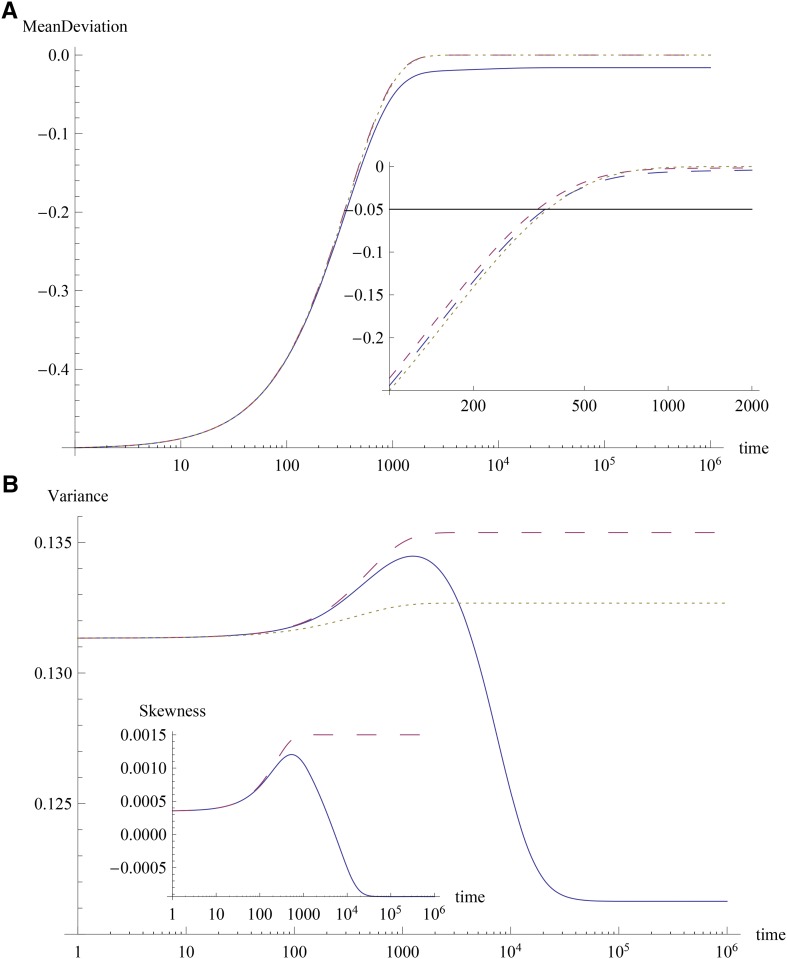
Response to change in optimum when most effects are small. The plot shows the results for (A) mean deviation $\Delta c_{1}\left(t\right)$ and (B) variance $c_{2}\left(t\right)$ and skewness $c_{3}\left(t\right)$ obtained using the exact numerical solution of the full model (solid) and the short-term dynamics model (large dashes). The dotted curves show the time-dependent solution (11) for mean and (A.1) for variance. The parameters are $\ell =50,s=0.02,\mu =5\times 10^{-5},\hat{\gamma }=0.14>\bar{\gamma }=0.05,z_{o}=-0.0012,z_{f}=0.5,n_{l}=5$. The effects are chosen from an exponential distribution, and the parameter C=−0.01 (see Appendix A). The inset in the top figure shows the difference $\Delta c_{1}\left(t\right)-\Delta c_{1}^{\text{*}}$ as a function of time for the full model when the effects are gamma-distributed with shape parameter *k* = 1 (large dashes), 5 (small dashes), and 20 (dotted).

The effects at most of the loci can be smaller than the scale $\hat{\gamma }$ either if *k* is large and $\bar{\gamma }<\hat{\gamma }$, or if *k* is small. Then for most loci, at short times, the full model defined by (4) can be approximated by

$$\frac{\partial p_{i}}{\partial t}=-s\gamma_{i}p_{i}q_{i}\Delta c_{1}$$ (10a)

$$\frac{\partial c_{n}}{\partial t}=-s\Delta c_{1}c_{n+1}\;,\;n\geq 1,$$ (10b)

where the last equation for cumulants is obtained from the results of Bürger (1991). Equation (10b) for n=1 shows that the magnitude of the mean deviation decreases with time, and for the phenotypic optimum smaller than the maximum attainable value of the mean $\left(z_{o}\ll \bar{\gamma }\ell \right)$, the trait mean becomes close to the phenotypic optimum at large times (see Figure 2A). We now assume that the variance $c_{2}$ is independent of time and stays at its initial value $c_{2}\left(0\right)$ (Chevin and Hospital 2008). As explained in Appendix A, this approximation is good when a combination of the initial cumulants is small (see also Figure 2B). This allows us to solve (10a) and (10b), and we immediately find that

$$\Delta c_{1}\left(t\right)=\Delta c_{1}\left(0\right)e^{-c_{2}\left(0\right)st}$$ (11)

$$p_{i}\left(t\right)=\frac{p_{i}\left(0\right)}{p_{i}\left(0\right)+q_{i}\left(0\right)e^{\frac{\gamma_{i}\Delta c_{1}\left(0\right)}{c_{2}\left(0\right)}\left(1-e^{-c_{2}\left(0\right)st}\right)}}\;.$$ (12)

Equation (11) shows that the mean deviation approaches zero over a time scale $t_{\times }∼{\left(sc_{2}\left(0\right)\right)}^{-1}$.

Next we analyze the long-term evolution of the allele frequencies. As Figure 2A shows, there is a small but nonzero mean deviation $\Delta c_{1}^{\text{*}}$ in the stationary state. Taking this into consideration and accounting for the other terms in (4), for $t>t_{\times }$, we can write

$$\frac{\partial p_{i}}{\partial t}=-\frac{s}{2}\gamma_{i}^{2}p_{i}q_{i}\left(1-2p_{i}+\frac{2\Delta c_{1}^{\text{*}}}{\gamma_{i}}\right)+\mu \left(1-2p_{i}\right).$$ (13)

For $\Delta c_{1}^{\text{*}}=0$, the aforementioned equation can be easily solved to give

$$p_{i}^{\left(\pm \right)}\left(t\right)=\frac{1}{2}\left(1\pm \sqrt{\frac{1-m_{i}}{1-M_{i}\left(t\right)}}\right)\;,t>t_{\times }\;,$$ (14)

where

$$M_{i}\left(t\right)=\frac{4p_{i}^{2}\left(t_{\times }\right)-4p_{i}\left(t_{\times }\right)+m_{i}}{{\left(2p_{i}\left(t_{\times }\right)-1\right)}^{2}}\;e^{-\frac{s\gamma_{i}^{2}\left(1-m_{i}\right)\left(t-t_{\times }\right)}{2}},t>t_{\times },$$ (15)

$m_{i}={\left(\hat{\gamma }/\gamma_{i}\right)}^{2}$ and $p_{i}\left(t_{\times }\right)$ is obtained from (12). We check that the stationary state solutions $\left(1\pm \sqrt{1-m_{i}}\right)/2$ and 1/2 are obtained from the above result for $m_{i}<1$ and >1, respectively. Furthermore, the solution $p_{i}^{\left(+\right)}\left(t\right)$ is obtained for $p_{i}\left(t_{\times }\right)>1/2$ and $p_{i}^{\left(-\right)}\left(t\right)$ for $p_{i}\left(t_{\times }\right)<1/2$.

**Figure 3:**
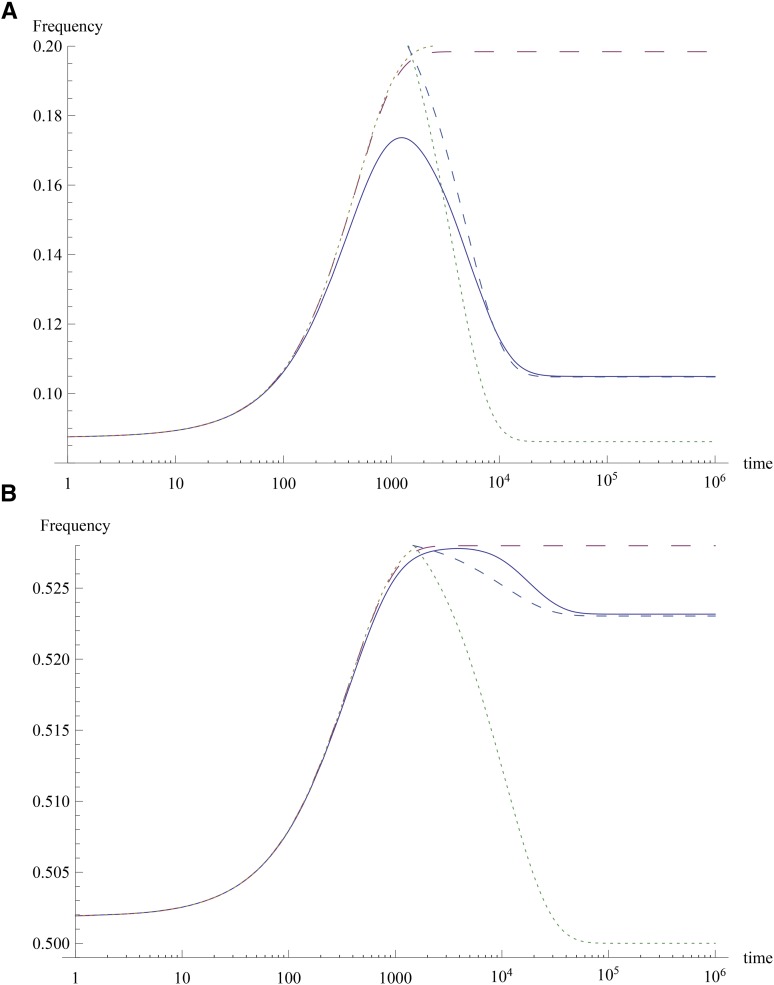
Response to change in optimum when most effects are small. The plot shows the allele frequencies for two representative loci with (A) $\gamma_{i}=0.252$ and (B) $\gamma_{i}=0.028$ for the full model (solid) and short-term dynamics model (large dashes). The dotted curves show the time-dependent solution (12) for $t<t_{\times }$ and (14) for $t>t_{\times }$ where $t_{\times }=1500$. The dashed curve for $t>t_{\times }$ is the solution of (13) with $\Delta c_{1}^{\text{*}}=-0.016$. The other parameter values are the same as in Figure 2.

Figure 2 and Figure 3 show a comparison between the numerical solution of (4) and the approximation described previously, when the initial condition is the stationary state of the population equilibrated to a phenotypic optimum *z*_0_. The initial mean deviation $\Delta c_{1}\left(0\right)$ is seen to be close to $-z_{f}$, and the initial variance $c_{2}\left(0\right)=c_{2}^{\text{*}}$ for the zero mean deviation is 0.0967, which is close to the value 0.131 obtained from the set of effects used in Figure 2 and Figure 3. As Figure 2 shows, the dynamics of the mean deviation are captured well by (11) and approach a stationary value close to zero $\left(\Delta c_{1}^{\text{*}}\approx -0.016\right)$ in about 1500 generations. The variance also evolves with time, but the change is not substantial and the approximation $c_{2}\left(t\right)\approx c_{2}\left(0\right)$ is good over the time scale directional selection toward the phenotypic optimum operates. Equation (11) also indicates that directional selection toward the optimum will occur faster when the initial variance is large since $t_{\times }∼1/c_{2}^{\text{*}}$.

Because the stationary genetic variance displays a nonmonotonic dependence on the shape parameter *k* of the gamma distribution (see Figure 1), the relaxation time for the mean deviation is expected to decrease and then increase with increasing *k*. Indeed, as the inset of Figure 2A shows, the difference $c_{1}\left(t\right)-c_{1}^{\text{*}}$ (which, by definition, is zero in the equilibrium state for all *k*) equals a reference value −0.05 at time 360, 340, and 370 for *k* = 1, 5, and 20, respectively.

The allele frequency dynamics are shown in Figure 3. We see that although the short-term dynamics can be accurately described by (12) for loci with effects smaller than or close to the distribution mean, there is a substantial difference when the effects are larger than the mean. This is because for such loci, the initial frequency is not close to half and the term involving *q_i_ − p_i_* on the RHS of (4) cannot be neglected. For $t>t_{\times }$, the long-term behavior described by (13) is shown with $\Delta c_{1}^{\text{*}}=0$ and the actual mean deviation.

### When most effects are large

When $\bar{\gamma }>\hat{\gamma }$ and *k* is large, the number of loci with large effects is also large, and the initial allele frequencies are close to either zero or one. In this parameter regime, both the variance and the skewness may change appreciably during directional selection toward the optimum, and the constant-variance approximation discussed above is not suitable. However, at very short times when Δ*c*_1_ (*t*) is close to its initial value, the solution (12) for the allele frequency gives

$$p_{i}\left(t\right)\approx \frac{1}{1+\frac{q_{i}\left(0\right)}{p_{i}\left(0\right)}e^{\gamma_{i}\Delta c_{1}\left(0\right)st}}\;.$$ (16)

From the aforementioned equation, we first note that the allele frequency at large-effect loci changes fast as expected intuitively. Equation (16) also shows that for Δ*c*_1_ (0) < 0, the allele frequency quickly increases toward unity, if the initial frequency is close to unity and therefore does not contribute to the dynamics of the variance or skewness. Thus, to understand the short-term dynamics, we need to focus our attention on large-effect loci with low initial allele frequency for negative initial mean deviation. Similar remarks apply to the situation when Δ*c*_1_ (0) is positive where the large-effect loci with high initial frequency determine the dynamics.

**Figure 4:**
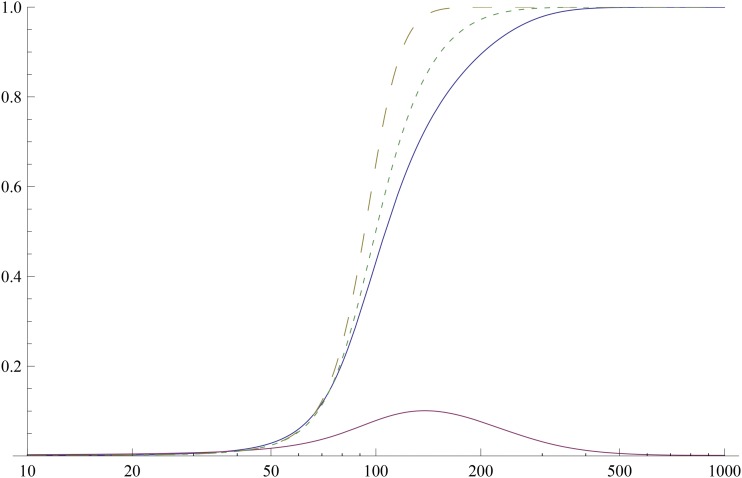
Response to change in optimum when most effects are large. The plot shows the exact numerical solution of the full model (solid) and the equations (20) (large dashes) and (21) (small dashes) for the dynamics of the allele frequency *P* with the largest effect and lowest initial frequency $\left(\text{Γ}=0.776,P_{0}=3.3\times 10^{-4}\right)$. The solid curve at the bottom shows the numerical solution of the full model for the frequency of the next relevant locus with effect size 0.319 and initial frequency $1.9\times 10^{-3}$. The parameters are $\ell =20,s=0.1,\mu =10^{-5},\hat{\gamma }\approx 0.028\ll \bar{\gamma }=0.2,z_{o}=7.8\times 10^{-5},z_{f}=1.5,n_{l}=19$.

In the following, we assume that Δ*c*_1_ (0) < 0 and consider the time evolution of the allele frequency *P* of the largest effect locus with lowest initial frequency. Figure 4 shows that the allele frequency *P* sweeps to fixation, but the frequency of the next relevant locus (*i.e.*, the next largest effect locus with low initial frequency) does not. In such a case, we can approximate the mean *c*_1_ and variance *c*_2_ by the contribution from the frequency *P* with effect Γ, and obtain

$$c_{1}\left(t\right)\approx 2\text{Γ}\left(P-P_{0}\right)+c_{1}\left(0\right)$$ (17a)

$$c_{2}\left(t\right)\approx 2{\text{Γ}}^{2}\left(PQ-P_{0}Q_{0}\right)+c_{2}\left(0\right),$$ (17b)

where $P_{0}\equiv P\left(0\right)$. Then using the aforementioned expression for the phenotypic mean in (4) and neglecting mutations (since most effects are large), we get

$$\frac{\partial P}{\partial t}=-s{\text{Γ}}^{2}P\left(1-P\right)\left(P+\alpha \right),$$ (18)

where

$$\alpha =\frac{\text{Γ}+2\Delta c_{1}\left(0\right)}{2\text{Γ}}-2P_{0}.$$ (19)

We thus find that the allele frequency *P* is a solution of the following equation:

$$\frac{{\left(P/P_{0}\right)}^{1+\alpha }}{{\left(Q/Q_{0}\right)}^{\alpha }}=e^{-s{\text{Γ}}^{2}\alpha \left(1+\alpha \right)t}\frac{P+\alpha }{P_{0}+\alpha }.$$ (20)

An explicit solution of (20) seems hard to obtain since *α* is in general not an integer. However, for large and negative *α*, the aforementioned equation yields

$$P=\frac{1}{1+\frac{Q_{0}}{P_{0}}e^{-s{\text{Γ}}^{2}\left|\alpha \right|t}}.$$ (21)

Thus for $z_{f}\gg \text{Γ}$, the frequency *P* sweeps to fixation in a time of order ${\left(s\text{Γ}z_{f}\right)}^{-1}$.

**Figure 5:**
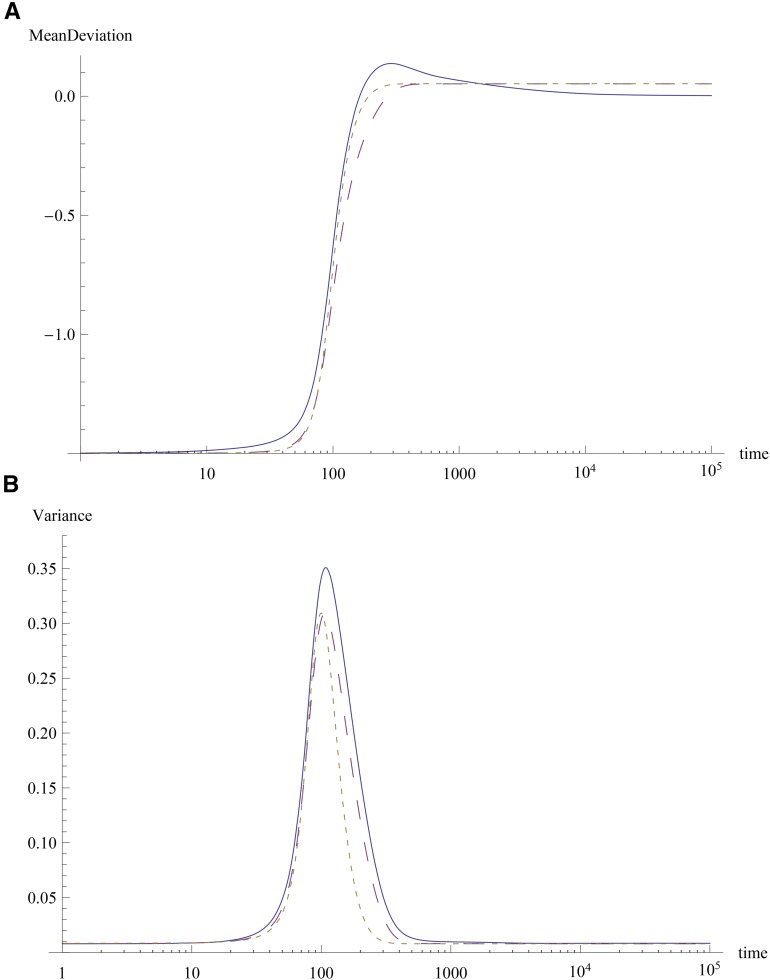
Response to change in optimum when most effects are large. Solid lines show the mean deviation (A) and variance (B), whereas the large dashed curves show the contribution to these cumulants from the locus with the largest effect and lowest initial frequency $\left(\text{Γ}=0.776,P_{0}=3.3\times 10^{-4}\right)$. In both cases, the exact numerical solution of the full model is used. The numerical solution of (18) (small dashes) is also shown. The other parameter values are the same as in Figure 4.

Figure 4 shows the allele frequency of the largest effect locus with lowest initial frequency obtained using (4). It agrees reasonably well with the solution of (20) and the expression (21) where *α* = −1.43. In Figure 5, the dynamics of the first two cumulants given by (1a) and (1b) are compared with the approximate expressions (17a) and (17b), respectively, and we see a good agreement.

A detailed numerical analysis of the set of parameter values of Figure 4 suggests that the dynamics of this example can be understood by considering one, two, or three of the largest-effect loci. When only the largest-effect locus was required, the second largest effect was much lower than the largest one.

## Discussion

One of the fundamental questions in adaptation is whether the adaptive process is governed by many loci of small effect or few loci of large effect (Orr, 2005). However, which effects are small, and which large? de Vladar and Barton (2014) have provided a scale $\hat{\gamma }∼\sqrt{\mu /s}$ for the size of effects, which is a function of basic population genetic parameters, namely mutation probability *μ* and selection coefficient *s*, relative to which an effect is defined as large or small. For a given distribution of effects, assumed here to be a gamma distribution with mean $\bar{\gamma }$ and shape parameter *k*, an effect is small (large) if it is below (above) $\hat{\gamma }$. But for fixed $\bar{\gamma }$ and $\hat{\gamma }$, whether most or a few effects are small depends on the shape parameter *k*: for small *k*, most effects are small, but for large *k*, the number of small effects depends on the ratio $\gamma_{r}=\hat{\gamma }/\bar{\gamma }$.

### Genetic variance in stationary state

Here we have provided analytical expressions for the stationary genetic variance $c_{2}^{\text{*}}$ when the effects are locus-dependent. We find that when most effects are small, $c_{2}^{\text{*}}$ is a nonmonotonic function of the shape parameter of the gamma distribution (going through a maximum for intermediate values of *k*; see Figure 1). In contrast, it increases monotonically when most effects are large. As Figure 1 shows, when the shape distribution is narrow, large (small) effects contribute most to the variance when $\gamma_{r}>1\left(<1\right)$. However, for broad distributions, although the number of small effects is large, small effects do not contribute much when $\gamma_{r}<1$. The HoC variance is obtained irrespective of *k* when $\gamma_{r}\to 0$ because all loci have large effect in this limit. As noted previously (de Vladar and Barton, 2014), HoC provides an upper bound on the genetic variance.

### Dynamics when most effects are small

As the distribution of quantitative trait loci measured in experimental and natural populations (Hayes and Goddard, 2001; Mackay, 2004; Visscher, 2008; Goddard and Hayes, 2009) find most effects to be small, it is important to study this situation in detail. Here we have obtained analytical expressions for the dynamics by assuming the genetic variance to be constant. Although the fact that the variance does not change much in time when most effects are small was observed numerically in de Vladar and Barton (2014), an explanation of this behavior was not provided. Here, as explained in Appendix A, it is a good approximation to assume the variance to be time-independent provided the product of the initial values of the mean deviation and skewness is small.

In the absence of mutations, Chevin and Hospital (2008) have considered the effect of background with a time-independent genetic variance on the frequency at a single focal locus. Their results match the ones obtained here using the short-term dynamics model with directional selection only; in particular, (11) and (12) match the results (21) and (25) of Chevin and Hospital (2008), respectively, on identifying their parameters $\omega^{2}$ and *a* with 1/s and *γ* from this study.

Our basic result concerning the dynamics of the phenotypic mean is that it relaxes over a time scale that is inversely proportional to the initial variance. Because the variance is of order *ℓ*, we thus have the important result that the mean approaches the optimum faster if a larger number of loci is involved. Moreover, this time depends nonmonotonically on the shape parameter of the gamma distribution. Note that the phenotypic mean deviation relaxes to zero when the phenotypic optimum is far below the upper bound $\bar{\gamma }\ell$ on the phenotypic mean. However, when the phenotypic optimum exceeds the maximum typical value of the mean, such that the mean deviation remains substantially different from zero at late times, (10b) shows that all higher cumulants vanish at the end of the phase of directional selection.

### Dynamics when most effects are large

When the initial mean deviation is moderately large (and negative), the genetic variance changes by a large amount over the time scale directional selection occurs and the dynamics can be understood by considering a few loci whose effect is large but initial frequency is low.

However, for larger mean deviations (but smaller than $\ell \bar{\gamma }$), a few large-effect loci do not completely capture the dynamics of the mean and the variance. As Supporting Information, Figure S1 shows, the initial increase of the absolute mean deviation and the transient rise of the variance can be explained by considering the large-effect locus. At later times, however, as the change in variance is small, we can use the constant-variance approximation to understand the dynamics of the phenotypic mean deviation until it nearly vanishes. The constant-variance approximation also can be used when the initial mean deviation is sufficiently small (see Figure S2).

### Applications

The approximations presented here hold for the short-term evolution of phenotypic traits and allele frequencies. This means that our results may be used to understand polygenic selection driving rapid adaptation. In this respect, our most important result is that the mean of a phenotypic trait may respond faster to a sudden environmental change when the number of loci is large and most effects are small.

Evidence for rapid phenotypic evolution has been reported in recent years from several groups of organisms. For instance in *Drosophila subobscura*, latitudinal clines of wing size have been formed within 20 years since this species colonized America (Huey *et al.*, 2000). Similarly, in field experiments in which lizard populations were newly established on small islands in the Bahamas, the hindlimbs adapted very quickly to the different vegetations on the islands (Kolbe *et al.*, 2012). To our knowledge, however, data from GWAS are not yet available in these cases.

The theory presented here can also be applied to the large amounts of GWAS data that have been gathered in model species such as humans and *Drosophila*. To analyze the observed allele frequency shifts in single-nucleotide polymorphisms associated with quantitative traits, such as human height (Turchin *et al.* 2012) and cold tolerance in *Drosophila* (Huang *et al.* 2012), the results derived in this study provide a more general theoretical basis than the dynamical equations used in previous analyses (*e.g.*, Turchin *et al.* 2012).

### Open questions

The analytical calculations in this article work when the phenotypic mean at the equilibrium coincides exactly with the optimum. However, in the stationary state, there is a small but nonzero mean deviation due to which the long-term dynamics are not accurately captured, especially when effects are small, as shown in Figure 3B. An improved calculation of the dynamics that takes a nonzero mean deviation into account is certainly of interest, for instance to estimate the frequency of selective fixations (leading to selective sweeps) in this model (Chevin and Hospital 2008; Pavlidis *et al.* 2012; Wollstein and Stephan 2014).

Another open question concerns the generality of our results presented here. The current model perhaps oversimplifies biological reality in that it neglects genetic drift and assumes additive effects, symmetric mutations and free recombination between loci. It can be shown that the current model (neglecting mutation) can be derived from the classical symmetric viability model with arbitrary position of the optimum under the quasi-linkage equilibrium assumption. In the latter model, the probability of selective fixation has been studied numerically for up to eight loci (Pavlidis *et al.* 2012; Wollstein and Stephan 2014). However, at present we are lacking an analytical understanding of the role of recombination in this model and how it relates to the high-recombination limit represented by our current model.

## Supplementary Material

Supporting Information

## Appendix

### A Validity of Constant-Variance Approximation

As explained in the main text, we obtain (11) when the variance is assumed to be constant in time. To see under what conditions this approximation holds, we find a correction to the variance by assuming that the skewness $c_{3}$ is nonzero and time-independent (see the inset of Figure 2B). Using (11) on the RHS of (10b) for n=2, we immediately get

$$c_{2}\left(t\right)=c_{2}\left(0\right)\;\left[1-C\left(1-e^{-c_{2}\left(0\right)st}\right)\right],$$ (A.1)

where $C=\Delta c_{1}\left(0\right)c_{3}\left(0\right)/c_{2}^{2}\left(0\right)$. Plugging the above solution into (10b) for n=1 gives the mean deviation as

$$\Delta c_{1}\left(t\right)=\Delta c_{1}\left(0\right)\;\exp\left[-stc_{2}\left(0\right)\left(1-C\right)-C\left(1-e^{-stc_{2}\left(0\right)}\right)\right].$$ (A.2)

The solution (11) is recovered if the constant C, which depends on the initial value of the first three cumulants, is negligible.

If we start with the initial condition in which the population is equilibrated to an optimum and most effects are small, since most initial allele frequencies are close to one half, the initial variance is substantial and the skewness is close to zero. In this case, the constant-variance approximation is expected to work well. However, if most effects are large, since most initial allele frequencies are close to fixation, although the skewness remains small, the variance also becomes small, thus leading to an increase in C. Then for sufficiently small mean deviations, we also may employ the constant-variance approximation when most effects are large.
