# Supplementary material for: Response of Polygenic Traits Under Stabilizing Selection and Mutation When Loci Have Unequal Effects
Source: G3 (Bethesda). 2015 Mar 31;5(6):1065–74. doi: 10.1534/g3.115.017970 (PMC4478537; doi:10.1534/g3.115.017970)
Supplement: Supporting Information [file supp_5_6_1065__index.html]

Response of Polygenic Traits Under Stabilizing Selection and Mutation When Loci Have Unequal Effects — Supporting Information 

# Response of Polygenic Traits Under Stabilizing Selection and Mutation When Loci Have Unequal Effects

## Supporting Information for Jain and Stephan, 2015

**Files in this Data Supplement:**

- Supporting Information - Figures S1-S2 (PDF, 195 KB)
- Figure S1 - Response to change in optimum when most effects are large. Solid lines show the mean deviation (1a) and variance (1b), while the large dashed curves show the contribution to these cumulants from the locus with the largest effect and lowest initial frequency. (PDF, 61 KB)
- Figure S2 - Response to change in optimum when most effects are large. Solid lines show the mean deviation (1a) and variance (1b), while the two dashed curves show the contribution to these cumulants from the first two relevant loci with effect 0.77 (large dashes) and 0.34 (small dashes). (PDF, 56 KB)
