# Supplementary material for: Response of Polygenic Traits Under Stabilizing Selection and Mutation When Loci Have Unequal Effects
Source: G3 (Bethesda). 2015 Mar 31;5(6):1065–74. doi: 10.1534/g3.115.017970 (PMC4478537; doi:10.1534/g3.115.017970)
Supplement: Supporting Information [file supp_g3.115.017970_FigureS1.pdf]

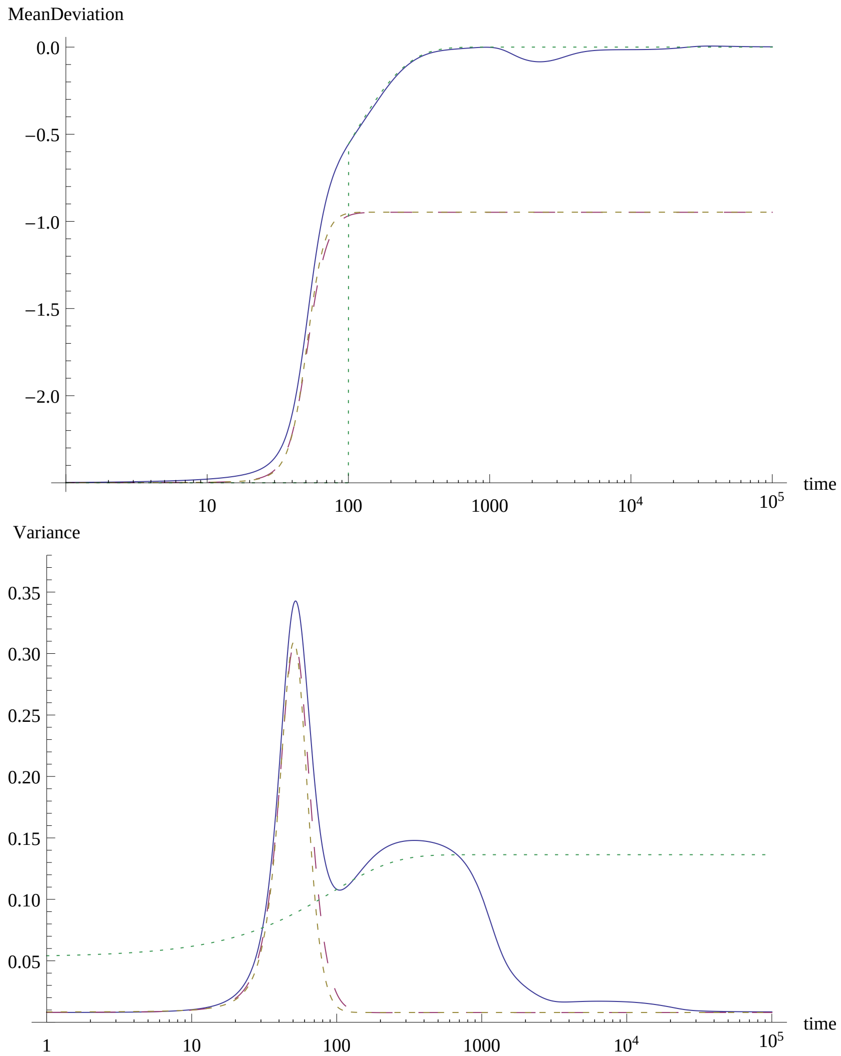

Figure S1: Response to change in optimum when most effects are large. Solid lines show the mean deviation (1a) and variance (1b), while the large dashed curves show the contribution to these cumulants from the locus with the largest effect and lowest initial frequency ($\Gamma = 0.776, P_0 = 3.3 \times 10^{-4}$). In both cases, the exact numerical solution of the full model is used. The numerical solution of (18) (small dashes) is also shown. The dotted curves show (11) and (A.1) for $t > 100$. The final optimum value $z_f = 2.5$ and the other parameter values are the same as in Fig. 4.
