# Supplementary material for: Response of Polygenic Traits Under Stabilizing Selection and Mutation When Loci Have Unequal Effects
Source: G3 (Bethesda). 2015 Mar 31;5(6):1065–74. doi: 10.1534/g3.115.017970 (PMC4478537; doi:10.1534/g3.115.017970)
Supplement: Supporting Information [file supp_g3.115.017970_FigureS2.pdf]

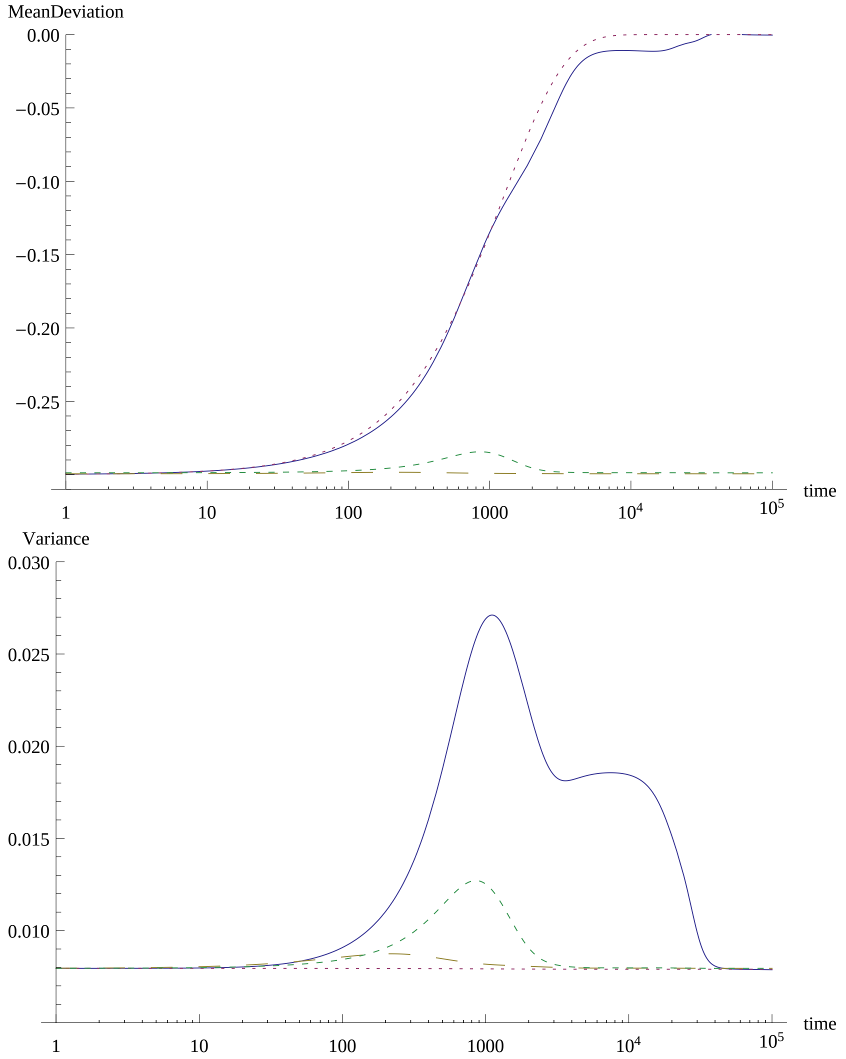

Figure S2: Response to change in optimum when most effects are large. Solid lines show the mean deviation (1a) and variance (1b), while the two dashed curves show the contribution to these cumulants from the first two relevant loci with effect 0.77 (large dashes) and 0.34 (small dashes). In both cases, the exact numerical solution of the full model is used. The dotted curves show (11) and (A.1) with $\mathcal{C} = 0.006$. The final optimum value $z_f = 0.3$ and the other parameter values are the same as in Fig. 4.
